# Care cascade of hypertension across stages among older adults in India

**DOI:** 10.1371/journal.pone.0335627

**Published:** 2025-12-03

**Authors:** Umakanta Sahoo, Suraj Maiti, Sanjay K. Mohanty

**Affiliations:** 1 Department of Statistics, Sambalpur University, Burla, Odisha, India; 2 Department of Population and Development, International Institute for Population Sciences, Mumbai, Maharashtra, India; 3 Department of Economics, Virginia Tech, Blacksburg, Virginia, United States of America; Indian Institute of Dalit Studies (IIDS), INDIA

## Abstract

Hypertension is now a common disease and the single largest risk factor for premature mortality in India. Hypertensive individuals are not homogenous and have varying risks to life. Although the number of studies on the care cascade of hypertension in India is increasing, no attempts have been made to estimate the prevalence and cascade of care across different stages of hypertension. This study estimates the prevalence, awareness, and treatment of hypertension by stages of hypertension among older adults in India. We analyzed data on 58,787 adults aged 45 years and above from the Longitudinal Ageing Study in India (LASI), Wave 1 (2017−18). Hypertension stages were categorized in accordance with the classification given by the Ministry of Health and Family Welfare, Government of India, and regrouped as per global classification. The age-sex adjusted prevalence, awareness, and treatment rates for different stages of hypertension were estimated. Multinomial logistic regression and the Erreygers’ Concentration Index were used to assess socioeconomic inequalities in hypertension care. We estimated the prevalence of pre-hypertension at 39.9%, stage 1 hypertension at 22.1% and stage 2 hypertension at 9.9%. Increasing age and body mass index were associated with a higher chance of hypertension, whereas living with spouses and children meant having lower odds of hypertension across all stages. The economic condition of the household, educational attainment, and social groups were not significant predictors of hypertension across the stages. The awareness and treatment of hypertension were low across all the stages. The Erreygers’ Concentration Index on awareness and treatment was pro-rich across all the stages of hypertension. A large proportion of hypertensive patients at the advanced stage remain undiagnosed and untreated and carry a higher risk of premature mortality. The awareness and treatment of hypertension are lower among the poorer and socially disadvantaged populations than their richer and more privileged counterparts across all stages.

## Introduction

Hypertension is the single largest risk factor of cardiovascular disease, leading to premature mortality and disability worldwide [1–3]. In 2017, an estimated 10.2 million adults were hypertensive, contributing to 8.6% of total DALY (GBD, 2017). In low- and middle-income countries (LMICs), hypertension has become a common household disease and is now a growing into an epidemic and a common household disease [4,5]. The prevalence and incidence of hypertension vary across and between countries, age, sex, and income groups [5–9]. Older adults (aged 60 years and above) are vulnerable to these diseases and often require medical attention [6].

The cascade of hypertension care, comprising hypertension awareness, treatment, and control, is considered critical for reducing morbidity and mortality associated with cardiovascular complications. The rule of halves suggests that half of those who are hypertensive should be aware of the disease, and half of those who are aware should have control of the disease [10]. However, despite increasing public health efforts to manage hypertension through preventive care and control in LMICs, significant gaps remain in the care cascade [9,11]. The cascade care of hypertension care has a strong socio-economic gradient [9,12]. Studies suggest the high and growing prevalence of hypertension by age [6]. The gap in the prevalence of hypertension across socio-economic groups has narrowed [13].

Hypertension awareness, treatment, and control (ATC) are low across study settings [14,37]. The ATC is lower among the poor, the less educated, and older adults [9,13,15]. A recent cross-country analysis found a high degree of disparities in hypertension prevalence and care cascades, emphasizing the urgent need for public health interventions in low- and middle-income settings [16].

The International Society for Hypertension (ISH) classifies blood pressure by stages/ grades: normal if Systolic Blood Pressure (SBP)<130 mmHg or Diastolic Blood Pressure (DBP)<85 mmHg, high normal if SBP is between 130–139 mmHg and/or DBP is 85–89 mmHg, grade 1 if SBP 140–159 mmHg and/or DBP is 90–99 and grade 2 if SBP>=160 mmHg and/or DBP>=100. Any BP reading with SBP > 140 or DBP > 80 is classified as hypertensive [17].

The Existing global literature categorizes hypertension into four broad stages: normal (non-hypertensive), pre-hypertensive stage, stage 1, and stage 2 based on systolic and diastolic readings. An alternative classification based on grades of hypertension also exists, where grade 1 is similar to stage 1, while grade 2 and grade 3 correspond to stage 2 [18]. A study from the USA, Canada, and England found that England had a higher prevalence of stage-1 hypertension (16.7%) and stage-2 hypertension (5.1%) compared to Canada (stage-1: 5.4%; stage-2: 1.3%) and USA (stage-1: 11%; stage-2: 2.7%) [19]. In a study done among adults in Malaysia, the pre-hypertensive stage was more prevalent than the other stages, and the risk factors of the pre-hypertensive stage, stage 1, and stage 2 were diabetes, rural residence, and lower socio-economic status [20]. A 2015 study found that unhealthy diet, irregular meals, obesity, smoking, and drinking alcohol were the independent predictors of stage 1 hypertension [21].

India, with a population of 1,417 million hypertensive individuals in 2024, is home to the largest number of hypertensive patients worldwide (1.28 billion). Older adults aged 45 years and above account for 28.6% in 2025 and are projected to increase to 40.0% by 2050 [22]. Hypertension is the single most common disease in India, disproportionately affecting older adults with early onset in developed countries. There is an increasing availability of literature on the prevalence, risk factors, and care cascade of hypertension in India, and it suggests a high prevalence and lower awareness, treatment, and control of hypertension in the country [9,13,23,24].

This paper was conceptualized with the following rationale. First, since the Ministry of Health and Family Welfare (MoHFW), Government of India, provides guidelines for identifying the population by the stages of hypertension, providing evidence on the prevalence and variations of hypertension by stages across the socioeconomic gradient would be a helpful exercise. Prior studies have analyzed hypertension without considering its stages. Second, the severity of hypertension, particularly for undiagnosed and untreated cases, poses severe health risks. Therefore, it is crucial to understand the extent of awareness and treatment by stages of hypertension to generate evidence for health planning. Third, the risk to life may be higher for those in the more advanced stages of hypertension. Finally, we did not find any study that has examined the variation in prevalence, awareness, and treatment (PAT) by stages of hypertension in India. In this context, this study examines the prevalence, awareness, and treatment by stages of hypertension among older adults in India.

## Materials and methods

### Data

**Fig 1:**
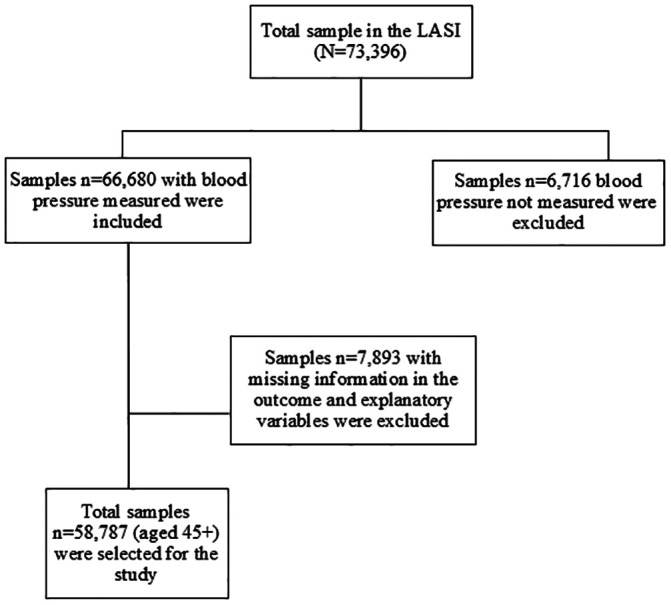
CONSORT diagram for analytic sample size, LASI wave-1 India, 2017−18.

The unit data from the Longitudinal Ageing Study in India (LASI), Wave 1, conducted during 2017−18 was used in the analyses. LASI is a nationally representative study on the health, economic, and social well-being of adults aged 45 years and above (and their spouses, irrespective of age). LASI used a random probability sampling technique, i.e., a multistage stratified sampling design in the selection of the sample households. It collected comprehensive information on housing, amenities, and the economic condition of households. Additionally, it collected detailed information on work, employment, retirement and pension status, chronic health conditions, functional health, mental health, extensive biomarkers, health care utilization, and health insurance for adults aged 45 years and above. The details of the sampling procedure, the instruments used, and the survey findings are available in the national report [25]. In this study, we excluded a sample of 6,716 older adults who did not have their blood pressure measured and a further sample of 7,893 older adults were also excluded who had missing information in the outcome and explanatory variables used in the analysis and finally, we used data on 58,787 older adults aged 45 years and above (complete case analytical sample) (**Fig 1**).

### Outcome variables

The stages of hypertension were computed from the measured blood pressure. Three blood pressure readings were recorded, with a one-minute gap between each. The average of the last two readings was used to calculate blood pressure readings. In literature, raised BP is defined as those with a systolic blood pressure of>= 140 mm Hg and/or diastolic blood pressure of>= 90 mm Hg or the use of medication for hypertension [26]. For our study, blood pressure was categorized by the threshold level of systolic blood pressure (SBP) and diastolic blood pressure (DBP), based on the guidelines given by the Government of India [18]. Individuals with SBP 140–159 (mmHg) or DBP 90–99 (mmHg) were labelled as grade 1 hypertensive, individuals with SBP 160–179 (mmHg) or DBP 100–109 (mmHg) were labelled as grade 2 hypertensive, and individuals with SBP>=180 (mmHg) or DBP>=110 (mmHg) were labelled as grade 3 hypertensive [18]. Broadly, grade 1 falls under stage 1, while grade 2 and grade 3 fall under stage 2 hypertension. Individuals at each stage who reported a hypertension diagnosis were classified as being “aware.” Individuals who were currently taking medicines to control BP were considered being “treated”. The classification system used by the Government of India is similar to the one used by the European Society for Hypertension/ European Society for Cardiology and International Society of Hypertension. Detailed cut-offs can be found in S1 Table.

### Explanatory variables

A set of potential socio-demographic confounders were considered in the analysis. These consisted of demographic characteristics, including age groups (“45-59”, “60-74”, and “75 and above”), sex (“male” and “female”), residence (“rural” and “urban”), and educational level (“no schooling”, “less than 5 years”, “5-9 years” and “10 or more years of schooling”), and social attributes, including caste (“Scheduled Tribe,” “Scheduled Caste,” “Other Backward Classes (OBC),” and “Others.”), religious affiliations (“Hindu,” “Muslim,” “Christian,” and “Others.”), marital status (“currently married,” “Widowed,” and “Others”), and living arrangement (“living alone,” “living with spouse and/or others,” “living with spouse and children,” and “living with children and/ or others”). Also considered were work status (“currently working,” “ever worked but not currently working,” and “never worked”) and BMI (“underweight,” “normal,” “overweight,” and “obese”). The monthly per capita consumption quintile (MPCE) (“poorest,” “poor,” “medium,” “richer,” and “richest”) was used as an economic variable. The remaining variables were tobacco consumption (“yes” and “no”), alcohol consumption (yes” and “no”), heart disease (“no” and “yes”), diabetes (“no” and “yes”), and health insurance coverage (“yes” and “no”).

### Statistical analysis

We computed the outcome variables, i.e., age- and sex-adjusted prevalence of hypertension, stratified by socio-demographic and health confounders. Age and sex adjustment was performed to standardize the prevalence estimates, and enable meaningful comparisons between groups while controlling for the effects of these confounders. Each estimate was reported with 95% confidence intervals (CI). All analyses were conducted using Stata version 16.0, accounting for the complex survey design and using appropriate survey weights.

The concentration index (CCI) was estimated to examine the socio-economic inequality in cascade care of hypertension. The standard concentration index, denoted below by C, is computed as twice the area between the concentration curve and the line of equality (the 45-degree line) [27]. The CCI is defined as:


$$C=\frac{\text{2}}{n\mu }\sum_{i=\text{1}}^{n}h_{i}R_{i}-\text{1}$$ (1)

where, μ is the mean of the health outcome variable${\;h}_{i}$ and $R_{i}$ is the rank of the individual in the MPCE quintile.

We used the concentration index with Erreygers’ correction, which is a quasi-absolute measure appropriate for binary health outcomes [28–29], and can be written as:


$$ECI=\frac{\text{4}\mu }{\left(b-a\right)}C.$$ (2)

where, C = standard concentration index as denoted in Equation 1 and *μ* is the mean of the health outcome variable with its range defined (*b* − *a*) (“b” is the upper bound, and “a” is the lower bound). A negative value of CCI suggests a concentration of the health outcome variables among those at the lower levels of socioeconomic status, whereas a positive value indicates concentration among the more affluent people. The concentration curve plotted the cumulative proportions of health variables against the cumulative proportions of the sample ranked by the equalized household wealth index (assets index).

Multinomial logistic regression was used to estimate the relative risk ratios (RRR) for various socioeconomic and demographic factors associated with different stages of hypertension, using pre-hypertensives as the reference category. The model can be expressed as:


$$P\left(Y_{i}=j|X_{i}\right)=\frac{e^{X_{i}\beta_{j}}}{\sum_{k=\text{1}}^{J}e^{X_{i}\beta_{k}}}$$ (3)

where Y_i_ is the outcome for the i^th^ individual, X_i_ is the vector of predictor variables, and $\beta_{\text{j}}$ is the vector of regression coefficients for the j^th^ outcome. We calculated average marginal effects (AME) to assess the impact of the covariates on the probability of awareness and treatment across hypertension stages.

## Results

### Sample characteristics of older adults

**Table 1** presents the sample characteristics by stages and grades of hypertension. The samples were categorized into Optimal, Pre-hypertensive, Stage 1 (grade 1), and Stage 2 (grade 2/ grade 3) individuals. Individuals aged 75 years and above constituted 16.5% of those with grade 2 hypertension and 14.1% of those with grade 3 hypertension, accounting for 14.6% in stage 2 hypertension. Grade 3 hypertension was higher among males (61.4%) compared to females (38.6%). Rural residents comprised a bigger sample (57.9%) in grade 3 hypertension compared to urban residents (42.1%). The richest MPCE quintile constituted a larger sample in grade 3 hypertension (34.0%) compared to the poorest MPCE quintile (14.8%). In stage 2, it was 23.2% for the richest MPCE quintile, whereas it was18.3% for the poorest MPCE quintile. A larger proportion of individuals are from the Hindu religion (83.2%) compared to other religious categories. In stage 2 hypertension, about 40% of older adults were overweight, and only 11.8% were underweight.

**Table 1:** Sample characteristics by stages of hypertension among older adults in India, 2017−18.

Socio-economic characteristics	Overall		Optimal		Pre-hypertensive		Stage 1 (Grade 1)		Grade 2		Grade 3		Stage 2 (Grade 2/ Grade 3)	
	%	N	%	N	%	N	%	N	%	N	%	N	%	N
Total	100	58787	100	15645	100	23231	100	13945	100	5272	100	694	100	5966
**Age**														
45-54	35.0	21888	41.4	6852	35.4	8971	29.5	4493	22.7	1352	45.5	220	26.3	1572
55-64	30.6	18242	30.7	4668	30.6	7257	30.3	4415	32.2	1679	23.8	223	31.9	1902
65-74	23.9	12983	19.4	2918	23.9	4984	28.4	3461	28.5	1454	16.6	166	27.2	1620
75+	10.6	5674	8.5	1207	10.2	2019	11.8	1576	16.5	787	14.1	85	14.6	872
**Sex**														
Male	46.2	27333	44.5	6739	46.9	11025	46.3	6786	46.3	2426	61.4	357	46.6	2783
Female	53.8	31454	55.5	8906	53.1	12206	53.7	7159	53.7	2846	38.6	337	53.4	3183
**Place of residence**														
Rural	70.7	38745	76.1	11215	69.4	15060	67.3	8598	69.1	3410	57.9	462	64.9	3872
Urban	29.3	20042	23.9	4430	30.6	8171	32.7	5347	30.9	1862	42.1	232	35.1	2094
**MPCE quintile**														
Poorest	20.4	10813	21.2	3022	20.2	4280	20.2	2419	19.7	960	14.8	132	18.3	1092
Poorer	20.2	11178	20.8	3122	19.9	4377	20.5	2585	19.8	977	17.0	117	18.3	1094
Middle	20.0	11297	19.6	3061	20.3	4363	20.1	2728	19.7	996	18.5	149	19.2	1145
Richer	20.0	12168	20.9	3188	19.7	4829	19.2	2901	21.5	1105	15.8	145	21.0	1250
Richest	19.4	13331	17.6	3252	19.9	5382	20.0	3312	19.4	1234	34.0	151	23.2	1385
**Caste**														
SC	19.7	9860	21.5	2898	19.0	3817	19.3	2248	18.0	799	16.5	98	15.0	897
ST	8.7	10448	8.4	2483	8.8	3946	8.6	2659	9.5	1173	9.4	187	22.8	1360
OBC	45.3	22361	46.6	6340	45.0	8848	43.9	5097	44.4	1842	53.7	234	34.8	2076
Others	26.3	16118	23.5	3924	27.1	6620	28.1	3941	28.1	1458	20.5	175	27.4	1633
**Religion**														
Hindu	82.5	43044	83.6	11995	83.1	17103	80.6	9936	80.6	3570	83.2	440	67.2	4010
Muslim	11.0	6825	10.5	1670	10.8	2717	12.5	1735	10.0	625	10.2	78	11.8	703
Christians	2.9	5915	3.1	1326	2.7	2309	2.8	1485	3.9	682	3.4	113	13.3	795
Others	3.5	3003	2.8	654	3.3	1102	4.1	789	5.5	395	3.2	63	7.7	458
**Education level**														
No education	50.9	27680	54.5	8041	49.3	10591	49.2	6193	51.7	2533	38.9	322	47.9	2855
Less than 5 years	11.2	6827	10.6	1800	11.3	2658	11.9	1676	10.7	613	12.0	80	11.6	693
5–9 years	20.6	13474	19.8	3434	21.0	5405	21.2	3267	20.8	1201	16.0	167	22.9	1368
10 years or more	17.3	10806	15.1	2370	18.4	4577	17.7	2809	16.8	925	33.1	125	17.6	1050
**Marital Status**														
Currently Married	74.1	44079	77.9	12298	74.8	17682	70.0	10058	67.7	3570	75.6	471	67.7	4041
Widowed	23.1	12798	18.6	2871	22.8	4779	27.4	3448	29.3	1516	20.9	184	28.5	1700
Others	2.9	1910	3.5	476	2.5	770	2.6	439	3.0	186	3.4	39	3.8	225
**Living Arrangement**														
Living alone	3.8	2093	2.5	393	4.0	818	4.4	580	5.3	267	3.5	35	5.1	302
Living with spouse and/or others	16.4	8970	16.6	2348	16.2	3531	16.4	2151	17.3	855	11.0	85	15.8	940
Living with spouse and children	56.7	34314	60.5	9762	57.5	13831	52.6	7709	49.6	2633	64.4	379	50.5	3012
Living with children and/or others	23.1	13410	20.5	3142	22.3	5051	26.6	3505	27.8	1517	21.1	195	28.7	1712
**Working Status**														
Currently working	47.5	27335	51.3	7792	47.4	11079	45.0	6138	39.2	2011	58.5	315	39.0	2326
Ever worked	26.8	15176	23.3	3645	26.3	5752	29.6	3903	34.9	1671	26.9	205	31.4	1876
Never worked	25.7	16276	25.5	4208	26.2	6400	25.4	3904	25.9	1590	14.6	174	29.6	1764
**Health Insurance**														
No	79.5	45301	80.2	11989	79.2	17818	79.5	10838	78.0	4120	81.4	536	78.0	4656
Yes	20.5	13486	19.8	3656	20.8	5413	20.5	3107	22.0	1152	18.6	158	22.0	1310
**BMI**														
Underweight	21.6	10806	32.1	4346	18.6	3745	15.7	1881	16.1	743	11.8	91	14.0	834
Normal	51.8	30817	51.5	8297	52.9	12447	51.8	7096	50.1	2624	37.8	353	49.9	2977
Overweight	19.8	12846	12.4	2245	21.3	5305	24.4	3698	24.3	1407	40.0	191	26.8	1598
Obese	6.7	4318	4.1	757	7.3	1734	8.2	1270	9.5	498	10.3	59	9.3	557
**Tobacco usage**														
Yes	38.1	21621	39.8	6112	37.5	8326	37.5	5040	37.1	1875	33.4	268	35.9	2143
No	61.9	37166	60.2	9533	62.5	14905	62.5	8905	62.9	3397	66.6	426	64.1	3823
**Alcohol usage**														
Yes	15.2	10572	14.1	2531	14.4	3986	17.0	2801	18.2	1075	16.5	179	21.0	1254
No	84.8	48215	85.9	13114	85.6	19245	83.0	11144	81.8	4197	83.5	515	79.0	4712
**Heart Disease**														
No	91.4	53952	91.4	14282	91.8	21383	90.6	12805	91.1	4848	90.8	634	91.9	5482
Yes	8.7	4835	8.6	1363	8.2	1848	9.4	1140	8.9	424	9.2	60	8.1	484
**Diabetes**														
No	88.4	51343	92.8	14350	87.6	20237	86.0	11807	81.7	4352	89.9	597	83.0	4949
Yes	11.6	7444	7.2	1295	12.4	2994	14.0	2138	18.3	920	10.1	97	17.0	1017

Refer to S1 Table.

**Fig 2:**
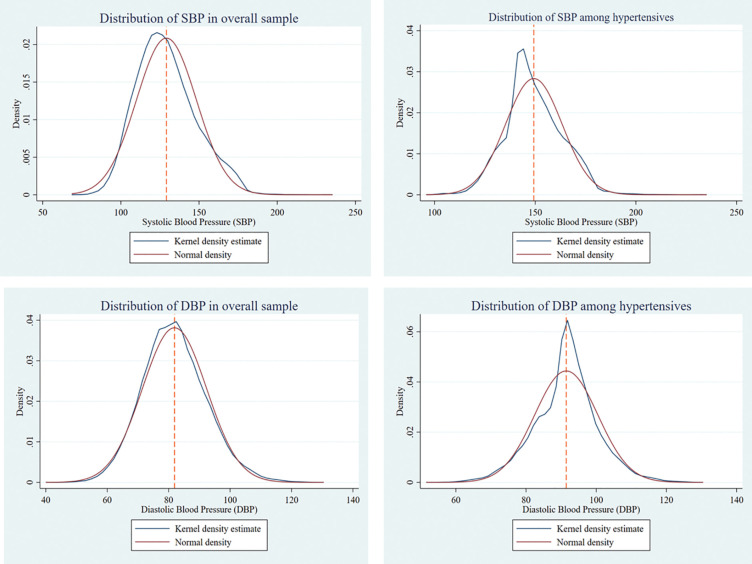
Distribution of SBP and DBP among all and hypertensive older adults in India, 2017−18.

**Fig 2** shows the distribution of blood pressure levels among older adults in India. The graph indicates that most of the older adults had blood pressure levels in the normal range, i.e., between 120–129 mmHg SBP and 80–84 mmHg DBP. Forty per cent of the older adults had blood pressure levels in the pre-hypertensive range (130–139 mmHg SBP or 85–89 mmHg DBP).

### Prevalence of hypertension by stages

The age-sex-adjusted prevalence of hypertension among older adults (aged 45 years and above) in India, stratified by hypertension stages, is presented in **Table 2**. The prevalence of pre-hypertension was the highest at 39.9% (95% CI: 39.2–40.7%), followed by grade 1 hypertension at 22.1% (95% CI: 21.3–22.9%), grade 2 hypertension at 7.8% (95% CI: 7.3–8.2%), and grade 3 hypertension at 1.1% (95% CI: 0.7–1.5%). The overall prevalence of stage 1 hypertension was 22.1%, while that of stage 2 was 8.9%. The prevalence of hypertension stages varied significantly with age, with stage 1 hypertension being higher among individuals aged 65–74 years (26.3%) compared to those aged 45–54 years (18.7%, p < 0.001). Grade 2 hypertension (a part of stage 2) also increased with advancing age, rising from 5.1% among individuals aged 45–54 years to 12.2% among those aged 75 years and above (p < 0.001). Prevalence rates showed no significant gender difference. However, urban residents exhibited a significantly higher prevalence of stage 1 hypertension (24.9%) compared to their rural counterparts (21.0%, p < 0.001), and a similar pattern was observed in the case of stage 2 hypertension, i.e., significantly higher in urban (9.9%) as compared to rural (8.5%, p < 0.015). In the case of religion categories, the prevalence of stage 1 hypertension was significantly higher among Muslims (25.2%) compared to Hindus (21.8%). However, the prevalence of stage (grade 2/grade 3) was higher in Christians compared to Hindus. Across all stages and grades of hypertension, the prevalence was higher among the older adults who had ever worked as compared to those who were currently working and those who had never worked. The prevalence of stage 1 (28.2%) and stage 2 (14.0%) hypertension was significantly higher among obese compared to underweight older adults (15.3% vs 5.9%).

**Table 2:** Age-sex adjusted estimates of prevalence of hypertension by stages among older adults in India, 2017−18.

Socio-economic characteristics	Pre-Hypertensive (n = 23,231)		Stage 1 (Grade 1) (n = 13,945)		Grade 2 (n = 5,272)		Grade 3 (n = 694)		Stage 2 (Grade 2/ Grade 3) (n = 5,966)	
	% (95% CI)	F-stat (p-value)	% (95% CI)	F-stat (p-value)	% (95% CI)	F-stat (p-value)	% (95% CI)	F-stat (p-value)	% (95% CI)	F-stat (p-value)
**Overall**	39.9 (39.2, 40.7)		22.1 (21.3, 22.9)		7.8 (7.3, 8.2)		1.1 (0.7, 1.5)		8.9 (8.4, 9.4)	
**Age**										
45-54	40.4 (38.8, 42.1)	0.57 (0.637)	18.7 (17.6, 19.8)	47.49 (<0.001)	5.1 (4.6, 5.5)	58.64 (<0.001)	1.45 (0.16, 2.74)	1.19 (0.312)	6.51 (5.28, 7.73)	14.73 (0.000)
55-64	39.9 (38.6, 41.2)		21.9 (20.8, 23.1)		8.2 (7.2, 9.2)		0.86 (0.68, 1.05)		9.06 (8.09, 10.03)	
65-74	40.0 (38.1, 41.8)		26.3 (25.0, 27.6)		9.3 (8.5, 10.1)		0.76 (0.57, 0.95)		10.07 (9.25, 10.89)	
75+	38.4 (36.0, 40.9)		24.6 (22.5, 26.8)		12.2 (10.7, 13.7)		1.44 (0.58, 2.30)		13.60 (11.86, 15.34)	
**Sex**										
Male	40.6 (39.6, 41.6)	2.71 (0.100)	22.0 (21.0, 23.0)	0.12 (0.723)	7.7 (7.1, 8.3)	0.17 (0.677)	1.47 (0.50, 2.44)	1.81 (0.179)	9.18 (8.22, 10.14)	0.82 (0.364)
Female	39.4 (38.4, 40.4)		22.2 (21.3, 23.1)		7.9 (7.4, 8.4)		0.79 (0.63, 0.95)		8.64 (8.10, 9.18)	
**Place of residence**										
Rural	39.2 (38.5, 39.9)	7.96 (0.005)	21.0 (20.3, 21.7)	10.99 (<0.001)	7.6 (7.1, 8.0)	1.74 (0.188)	0.91 (0.73, 1.09)	1.07 (0.301)	8.48 (7.94, 9.01)	5.98 (0.015)
Urban	41.8 (40.1, 43.4)		24.9 (22.6, 27.2)		8.4 (7.3, 9.4)		1.55 (0.31, 2.78)		9.91 (8.89, 10.94)	
**MPCE quintile**										
Poorest	39.5 (38.0, 41.0)	1.000 (0.408)	21.8 (20.3, 23.2)	1.38 (0.237)	7.4 (6.4, 8.4)	0.49 (0.742)	0.81 (0.59, 1.03)	0.62 (0.648)	8.21 (7.21, 9.21)	1.03 (0.392)
Poorer	39.2 (37.8, 40.6)		22.3 (21.2, 23.5)		7.6 (6.8, 8.3)		0.92 (0.45, 1.39)		8.49 (7.65, 9.34)	
Middle	40.7 (39.2, 42.1)		22.1 (20.9, 23.3)		7.7 (6.8, 8.5)		1.05 (0.71, 1.39)		8.71 (7.84, 9.57)	
Richer	39.4 (37.8, 41.0)		21.3 (19.6, 22.9)		8.4 (7.3, 9.5)		0.88 (0.64, 1.11)		9.26 (8.16, 10.37)	
Richest	41.0 (39.5, 42.5)		23.1 (21.3, 24.9)		8.0 (6.9, 9.0)		1.86 (0.01, 3.71)		9.86 (8.08, 11.64)	
**Caste**										
SC	38.5 (36.9, 40.2)	2.63 (0.048)	21.8 (20.5, 23.1)	2.63 (0.049)	7.2 (6.5, 7.9)	2.43 (0.063)	0.94 (0.63, 1.25)	1.1 (0.347)	8.13 (7.29, 8.98)	1.9 (0.128)
ST	40.5 (38.1, 42.8)		21.9 (20.1, 23.7)		8.7 (7.6, 9.8)		1.17 (0.84, 1.50)		9.88 (8.66, 11.10)	
OBC	39.7 (38.4, 41.0)		21.5 (20.1, 22.9)		7.6 (6.9, 8.3)		1.30 (0.39, 2.20)		8.90 (8.07, 9.73)	
Others	41.3 (40.1, 42.5)		23.5 (22.4, 24.6)		8.3 (7.5, 9.0)		0.87 (0.66, 1.08)		9.12 (8.37, 9.87)	
**Religion**										
Hindu	40.2 (39.4, 41.1)	1.18 (0.316)	21.6 (20.7, 22.5)	5.68 (<0.001)	7.6 (7.1, 8.1)	9.25 (<0.001)	1.10 (0.58, 1.62)	0.33 (0.801)	8.70 (8.13, 9.27)	7.74 (0.000)
Muslim	39.1 (36.6, 41.7)		25.2 (23.2, 27.2)		7.1 (6.0, 8.2)		1.07 (0.69, 1.44)		8.18 (7.02, 9.34)	
Christians	37.3 (31.4, 43.2)		21.3 (18.0, 24.6)		10.7 (8.7, 12.6)		1.42 (0.71, 2.13)		12.12 (10.08, 14.16)	
Others	37.8 (35.0, 40.5)		25.5 (22.9, 28.0)		12.1 (10.0, 14.3)		0.99 (0.48, 1.50)		13.13 (10.75, 15.51)	
**Education level**										
No education	38.8 (37.8, 39.7)	2.98 (0.030)	20.8 (20.0, 21.6)	6.21 (<0.001)	7.6 (7.0, 8.1)	0.98 (0.403)	0.92 (0.70, 1.13)	0.61 (0.609)	8.50 (7.92, 9.07)	2.25 (0.080)
Less than 5 years	40.3 (38.2, 42.4)		23.6 (22.0, 25.2)		7.3 (6.3, 8.3)		1.11 (0.38, 1.84)		8.45 (7.21, 9.69)	
5–9 years	40.6 (39.1, 42.1)		23.4 (22.2, 24.7)		8.3 (7.4, 9.3)		0.84 (0.63, 1.05)		9.17 (8.14, 10.19)	
10 years or more	42.3 (40.2, 44.5)		23.5 (20.2, 26.9)		8.2 (6.6, 9.7)		1.82 (0.21, 3.42)		10.11 (8.78, 11.45)	
**Marital Status**										
Currently Married	40.1 (39.2, 41.1)	1.75 (0.175)	21.3 (20.4, 22.2)	7.69 (<0.001)	7.5 (6.9, 8.1)	1.89 (0.152)	1.07 (0.58, 1.57)	0.32 (0.727)	8.58 (7.91, 9.26)	1.97 (0.140)
Widowed	40.0 (38.3, 41.7)		24.7 (23.2, 26.2)		8.4 (7.6, 9.2)		1.18 (0.60, 1.77)		9.60 (8.75, 10.45)	
Others	34.3 (28.4, 40.2)		21.3 (17.1, 25.5)		8.8 (6.7, 11.0)		1.44 (0.63, 2.25)		10.27 (7.96, 12.58)	
**Living Arrangement**										
Living alone	43.1 (39.1, 47.1)	1.59 (0.191)	24.0 (21.2, 26.8)	5.23 (0.001)	9.2 (7.5, 10.9)	1.59 (0.191)	1.20 (0.51, 1.89)	0.71 (0.544)	10.43 (8.61, 12.24)	1.53 (0.205)
Living with spouse and/or others	39.5 (37.1, 41.9)		21.5 (19.8, 23.2)		7.8 (6.8, 8.7)		0.85 (0.53, 1.17)		8.66 (7.69, 9.62)	
Living with spouse and children	40.3 (39.1, 41.4)		21.2 (20.3, 22.2)		7.4 (6.7, 8.1)		1.14 (0.57, 1.72)		8.57 (7.74, 9.40)	
Living with children and/or others	38.9 (37.3, 40.6)		24.3 (22.7, 25.9)		8.3 (7.5, 9.1)		1.15 (0.66, 1.64)		9.47 (8.66, 10.28)	
**Working Status**										
Currently working	39.3 (38.3, 40.4)	4.35 (0.013)	21.8 (20.7, 22.8)	1.59 (0.203)	6.9 (6.3, 7.6)	8.48 (<0.001)	1.19 (0.62, 1.76)	2.72 (0.066)	8.13 (7.34, 8.92)	8.7 (0.000)
Ever worked	39.4 (38.0, 40.8)		23.0 (21.8, 24.1)		9.0 (8.3, 9.7)		1.19 (0.77, 1.60)		10.20 (9.39, 11.01)	
Never worked	41.7 (40.4, 43.1)		21.9 (20.3, 23.4)		7.9 (7.0, 8.8)		0.78 (0.47, 1.10)		8.74 (7.92, 9.56)	
**Health Insurance**										
No	39.8 (39.0, 40.7)	0.33 (0.563)	22.1 (21.2, 23.0)	0.13 (0.714)	7.6 (7.1, 8.1)	4.23 (0.040)	1.13 (0.56, 1.70)	0.15 (0.701)	8.72 (8.11, 9.33)	1.88 (0.171)
Yes	40.4 (38.8, 42.1)		22.3 (21.1, 23.5)		8.6 (7.7, 9.5)		1.00 (0.76, 1.24)		9.58 (8.60, 10.56)	
**BMI**										
Underweight	34.5 (32.8, 36.2)	17.87 (<0.001)	15.3 (14.4, 16.3)	68.74 (<0.001)	5.3 (4.7, 5.9)	25.18 (<0.001)	0.59 (0.39, 0.78)	2.85 (0.036)	5.88 (5.25, 6.52)	45.82 (0.000)
Normal	40.6 (39.7, 41.6)		22.1 (21.2, 22.9)		7.5 (7.0, 8.0)		0.80 (0.64, 0.96)		8.34 (7.81, 8.86)	
Overweight	42.9 (41.4, 44.5)		28.1 (26.1, 30.2)		10.3 (9.1, 11.4)		2.21 (0.49, 3.92)		12.50 (10.91, 14.09)	
Obese	43.3 (38.1, 48.5)		28.2 (24.6, 31.7)		12.0 (9.7, 14.3)		2.05 (0.40, 3.70)		13.97 (11.12, 16.82)	
**Tobacco usage**										
Yes	38.9 (37.8, 40.0)	4.89 (0.027)	21.4 (20.4, 22.4)	2.24 (0.135)	7.3 (6.7, 8.0)	2.36 (0.124)	0.82 (0.58, 1.07)	0.99 (0.320)	8.12 (7.40, 8.85)	4.69 (0.030)
No	40.6 (39.6, 41.5)		22.6 (21.4, 23.8)		8.1 (7.4, 8.8)		1.33 (0.45, 2.20)		9.40 (8.63, 10.17)	
**Alcohol usage**										
Yes	36.9 (35.4, 38.5)	15.54 (<0.001)	25.2 (23.8, 26.5)	18.6 (<0.001)	9.8 (8.6, 11.0)	13.08 (<0.001)	0.94 (0.60, 1.29)	0.19 (0.663)	10.61 (9.33, 11.89)	6.64 (0.010)
No	40.5 (39.7, 41.3)		21.6 (20.7, 22.5)		7.4 (7.0, 7.9)		1.14 (0.53, 1.75)		8.59 (7.99, 9.18)	
**Heart Disease**										
No	40.1 (39.4, 40.9)	3.84 (0.050)	21.9 (21.1, 22.8)	3.1 (0.079)	7.8 (7.3, 8.2)	0.05 (0.826)	1.09 (0.62, 1.56)	0.24 (0.623)	8.86 (8.35, 9.38)	0.19 (0.666)
Yes	37.9 (35.8, 40.0)		24.1 (21.9, 26.4)		7.9 (6.5, 9.4)		1.28 (0.58, 1.98)		9.21 (7.67, 10.76)	
**Diabetes**										
No	39.6 (38.9, 40.4)	2.07 (0.151)	21.6 (20.8, 22.4)	15.55 (<0.001)	7.3 (6.8, 7.7)	11.21 (<0.001)	1.11 (0.62, 1.60)	0.05 (0.819)	8.38 (7.84, 8.92)	10.27 (0.001)
Yes	42.3 (38.9, 45.8)		25.8 (23.6, 27.9)		11.5 (9.1, 13.8)		1.05 (0.69, 1.40)		12.58 (10.20, 14.96)	

Refer to SM Table 1.

### Awareness and treatment by stages of hypertension

**Table 3** presents the age-sex adjusted estimates of awareness and treatment of hypertension among older adults in India. Among those with grade 2 hypertension, 44.5% were aware [95% CI: 41.6–47.3], and 38.3% were receiving treatment [95% CI: 35.4–41.2]. Among those with grade 3 hypertension, 42.8% were aware [95% CI: 23.9–61.7], and 39.3% were being treated [95% CI: 21.6–57.1] treated. The stage 1/grade 1 hypertension corresponds with an awareness rate of 34% [95% CI: 31.8–35.2] and a treatment level of 29% [95% CI: 27.0–30.2]. In the 65–74 age group, those with stage 1/ grade 1 hypertension had the highest awareness at 39.7%. Those aged 75 + had awareness and treatment rates of 39% and 32%, respectively, compared to early age of older adults (i.e., 45–54 years). Females had higher awareness and treatment rates than males across all grades/stages (e.g., 37%, 48%, and 48% awareness rates, respectively, for grades 1, 2, and 3 among females). Urban residents were more aware than rural residents (e.g., stage 1/ grade 1: 44% vs. 29%). Scheduled Tribes (STs) had the lowest awareness. Education correlated with greater awareness (e.g., 47%, 56%, and 46% for grades 1, 2, and 3 among those with ≥10 years vs. 27%, 39%, and 38% with no formal education). Among those with grade 3 hypertension, the rate of awareness was higher among those who had ever worked than those who were currently working (57% vs. 35%). Obese individuals consistently showed higher awareness and treatment. Non-users of tobacco/ alcohol and those with heart disease/ diabetes also had higher awareness and treatment rates across all grades.

**Table 3:** Age-sex adjusted estimates of awareness and treatment of hypertension by stages among older adults in India, 2017−18.

Socio-economic characteristic	Stage 1 (Grade 1)				Grade 2				Grade 3				Stage 2 (Grade 2/ Grade 3)			
	Awareness (%)	95% CI	Treatment (%)	95% CI	Awareness (%)	95% CI	Treatment (%)	95% CI	Awareness (%)	95% CI	Treatment (%)	95% CI	Awareness (%)	95% CI	Treatment (%)	95% CI
**Overall**	33.5	(31.8, 35.2)	28.7	(27.0, 30.2)	44.5	(41.6, 47.3)	38.3	(35.4, 41.2)	42.8	(23.9, 61.7)	39.3	(21.6, 57.1)	44.3	(40.7, 47.8)	38.4	(34.0, 41.9)
**Age**																
45-54	26.2	(23.4, 28.6)	22.1	(19.9, 24.2)	40.4	(36.0, 44.5)	312.1	(28.2, 35.7)	28.9	(4.0, 54.1)	27.6	(3.8, 51.3)	38.0	(30.2, 45.8)	31.1	(24.5, 37.3)
55-64	32.3	(30.1, 34.5)	27..8	(25.9, 30.0)	44.6	(37.9, 51.1)	40.0	(32.5, 46.8)	48.3	(37.6, 59.1)	44.1	(33.4, 55.0)	44.9	(38.8, 51.0)	40.3	(33.6, 46.7)
65-74	39.7	(37.0, 42.5)	34.8	(32.2, 37.5)	45.7	(41.9, 49.5)	49.7	(36.0, 43.7)	57.0	(44.0, 69.9)	50.0	(36.6, 63.5)	46.5	(42.6, 50.3)	40.5	(36.1, 44.2)
75+	39.2	(34.8, 43.4)	32.2	(28.0, 36.2)	47.5	(40.3, 55.1)	41.3	(34.1, 48.0)	60.8	(30.1, 90.4)	56.0	(23.2, 88.9)	49.1	(40.7, 57.5)	42.9	(34.2, 51.5)
**Sex**																
Male	29.3	(27.3, 31.2)	24.4	(22.8, 26.2)	39.8	(35.7, 43.8)	34.1	(30.2, 38.1)	38.7	(19.1, 54.0)	34.5	(16.4, 52.7)	40.0	(35.0, 44.5)	34.2	(29.8, 38.9)
Female	37.1	(34.9, 39.3)	32.2	(30.2, 34.3)	48.4	(45.3, 51.6)	42.0	(38.8, 45.2)	48.2	(27.1, 69.4)	45.8	(25.2, 66.4)	48.5	(45.1, 52.0)	42.4	(38.9, 45.9)
**Place of residence**																
Rural	28.8	(26.9, 30.6)	24.0	(22.3, 25.6)	40.5	(38.0, 43.1)	34.5	(31.9, 37.1)	40.4	(22.1, 58.8)	37.0	(19.8, 54.2)	40.9	(37.5, 44.3)	35.1	(31.9, 38.4)
Urban	43.1	(40.1, 46.1)	38.3	(35.4, 41.2)	53.4	(47.6, 59.1)	46.9	(40.8, 52.8)	48.0	(26.1, 70.0)	44.8	(24.1, 65.5)	51.7	(45.4, 58.0)	45.8	(39.6, 52.1)
**MPCE quintile**																
Poorest	28.3	(25.5, 31.1)	23.3	(20.7, 25.8)	39.8	(34.7, 44.8)	34.0	(28.7, 39.3)	31.4	(13.6, 49.2)	27.8	(11.3, 43.1)	38.5	(33.1, 43.7)	32.9	(27.4, 38.5)
Poorer	32.0	(28.5, 35.4)	27.4	(24.1, 30.6)	41.2	(36.7, 45.7)	34.1	(29.7, 38.5)	46.5	(24.3, 68.6)	42.2	(21.5, 64.0)	42.9	(37.6, 48.1)	36.0	(31.0, 41.1)
Middle	35.1	(32.0, 38.1)	29.4	(26.6, 32.3)	40.3	(35.3, 45.0)	34.4	(29.9, 39.0)	43.4	(22.5, 64.4)	40.4	(20.1, 59.9)	40.7	(35.4, 46.0)	35.1	(30.0, 40.2)
Richer	35.8	(33.1, 38.4)	31.4	(29.0, 33.9)	49.5	(43.5, 55.3)	43.9	(38.1, 49.7)	48.3	(25.8, 70.8)	44.3	(23.0, 65.4)	49.9	(44.0, 55.6)	44.2	(38.4, 50.0)
Richest	36.3	(33.0, 39.7)	31.9	(28.8, 35.0)	51.4	(45.8, 57.0)	45.0	(39.3, 50.5)	43.6	(21.0, 66.2)	41.0	(19.5, 62.5)	48.9	(42.4, 55.5)	43.4	(37.3, 49.6)
**Caste**																
SC	31.4	(28.5, 34.3)	26.0	(23.2, 28.8)	44.3	(39.3, 49.4)	37.5	(32.3, 42.6)	40.2	(20.5, 60.0)	37.8	(18.7, 56.8)	44.2	(38.5, 50.0)	38.0	(32.0, 43.9)
ST	19.6	(16.3, 23.1)	16.1	(12.9, 19.5)	28.9	(22.8, 35.2)	22.9	(17.8, 27.9)	33.0	(15.0, 50.9)	27.8	(11.2, 44.2)	29.6	(23.9, 35.4)	23.5	(18.4, 28.2)
OBC	33.0	(30.4, 35.6)	28.3	(26.0, 30.7)	43.6	(38.5, 48.6)	37.8	(32.7, 43.0)	44.2	(23.5, 65.0)	43.2	(22.6, 63.3)	43.4	(38.1, 48.8)	38.7	(33.3, 43.6)
Others	39.7	(37.3, 42.2)	34.7	(32.4, 37.0)	51.2	(47.3, 55.1)	44.9	(40.8, 48.9)	46.9	(24.7, 69.1)	39.2	(19.7, 58.9)	50.7	(46.4, 55.2)	44.0	(39.8, 48.5)
**Religion**																
Hindu	32.3	(30.5, 34.1)	27.4	(25.8, 30.0)	42.6	(40.0, 45.4)	36.6	(33.9, 39.4)	42.1	(23.2, 60.9)	38.5	(20.9, 56.1)	42.5	(38.9, 46.1)	36.8	(33.6, 40.3)
Muslim	38.9	(34.2, 43.6)	34.1	(29.9, 38.3)	50.5	(41.9, 59.1)	43.2	(34.4, 52.1)	51.2	(24.0, 78.4)	47.9	(22.1, 73.6)	51.3	(42.9, 59.6)	45.0	(35.9, 52.7)
Christians	31.7	(25.6, 37.7)	26.5	(21.3, 32.3)	47.9	(37.5, 58.1)	45.0	(35.0, 55.0)	36.8	(14.2, 59.5)	35.9	(12.9, 58.9)	46.6	(37.0, 56.1)	43.7	(34.3, 53.0)
Others	40.5	(34.0, 46.7)	36.8	(31.0, 43.1)	57.9	(49.7, 66.1)	49.5	(41.2, 57.1)	37.6	(12.8, 62.4)	34.0	(9.1, 58.1)	56.1	(48.8, 63.4)	47.9	(40.7, 55.0)
**Education level**																
No education	27.0	(25.3, 29.0)	22.4	(20.9, 24.3)	38.2	(34.5, 41.7)	31.9	(28.2, 35.4)	39.1	(20.8, 57.3)	36.9	(19.4, 54.8)	38.2	(34.3, 42.8)	32.3	(28.76.1)
Less than 5 years	34.9	(30.9, 38.8)	30.0	(25.9, 33.3)	43.5	(37.4, 49.7)	36.8	(30.8, 42.7)	50.5	(24.8, 76.3)	43.6	(20.9, 66.3)	45.8	(40.2, 52.9)	39.1	(32.6, 45.6)
5–9 years	38.1	(34.9, 41.3)	34.3	(31.2, 37.5)	51.1	(47.0, 56.1)	45.4	(41.1, 49.3)	40.5	(20.4, 60.7)	36.2	(18.4, 54.7)	50.4	(45.6, 55.1)	44.4	(39.9, 49.1)
10 years or more	46.9	(43.9, 49.9)	40.8	(37.7, 43.9)	56.5	(50.2, 62.7)	51.9	(45.5, 58.4)	46.0	(24.7, 74.3)	46.5	(22.7, 70.3)	54.4	(47.2, 61.6)	50.5	(43.3, 57.5)
**Marital Status**																
Currently Married	33.8	(31.5, 35.9)	29.0	(27.0, 31.0)	45.2	(41.3, 49.0)	40.0	(35.4, 43.3)	37.8	(20.4, 55.2)	35.6	(19.0, 52.3)	44.5	(40.3, 48.8)	38.9	(34.8, 43.7)
Widowed	33.8	(30.9, 36.6)	28.5	(26.0, 31.1)	42.8	(38.7, 46.9)	36.4	(32.5, 40.3)	54.0	(29.7, 78.3)	46.6	(24.6, 68.5)	44.0	(39.4, 48.4)	37.6	(33.5, 41.9)
Others	23.7	(17.1, 30.3)	22.3	(16.1, 28.5)	44.7	(32.0, 57.4)	36.0	(23.5, 48.5)	55.1	(24.3, 86.0)	54.0	(24.1, 84.1)	45.7	(34.2, 57.3)	38.0	(26.1, 49.5)
**Living Arrangement**																
Living alone	29.9	(24.5, 35.3)	25.9	(20.8, 31.1)	41.0	(31.6, 50.3)	34.0	(25.5, 42.5)	57.4	(26.3, 88.4)	49.0	(21.0, 78.9)	42.2	(33.4, 51.3)	35.2	(27.1, 43.4)
Living with spouse and/or others	32.9	(29.5, 36.6)	28.3	(24.7, 32.1)	41.9	(36.7, 46.9)	37.5	(31.8, 42.3)	42.6	(21.1, 63.9)	41.4	(20.4, 62.4)	41.3	(36.1, 46.5)	36.7	(32.0, 42.2)
Living with spouse and children	34.1	(31.8, 36.3)	29.2	(27.0, 31.3)	46.7	(41.6, 51.1)	40.1	(35.0, 45.1)	36.8	(19.5, 54.1)	34.2	(17.8, 50.6)	45.3	(40.4, 50.1)	39.8	(34.7, 44.7)
Living with children and/or others	33.4	(30.6, 36.1)	28.3	(25.7, 30.9)	43.5	(39.6, 47.5)	37.0	(33.3, 40.7)	53.2	(29.6, 76.8)	47.2	(25.4, 69.1)	44.5	(40.5, 48.9)	38.3	(34.3, 42.3)
**Working Status**																
Currently working	24.5	(22.2, 27.1)	20.4	(18.2, 22.5)	35.1	(31.1, 39.1)	29.3	(25.5, 33.0)	35.3	(17.9, 53.2)	32.5	(15.7, 49.1)	35.0	(30.6, 39.4)	29.6	(25.5, 33.7)
Ever worked	38.5	(35.5, 41.3)	32.9	(30.1, 35.6)	48.3	(44.1, 52.4)	41.2	(37.3, 45.3)	57.8	(33.8, 81.7)	53.1	(30.2, 75.9)	49.4	(45.0, 53.9)	42.6	(38.4, 46.9)
Never worked	42.8	(40.2, 46.0)	37.5	(34.3, 40.7)	53.3	(47.7, 58.9)	47.8	(42.1, 53.5)	39.6	(19.4, 59.6)	37.4	(18.0, 56.8)	52.2	(46.3, 58.0)	46.8	(40.9, 52.7)
**Health Insurance**																
No	33.0	(31.6, 35.0)	28.7	(26.8, 29.9)	44.9	(42.0, 47.7)	38.5	(35.7, 41.5)	41.6	(22.8, 60.3)	38.4	(20.8, 56.0)	44.8	(40.1, 48.1)	38.5	(34.9, 42.1)
Yes	35.4	(32.5, 37.6)	31.0	(28.0, 33.0)	43.2	(37.9, 48.4)	37.4	(32.1, 42.7)	46.8	(25.2, 68.4)	42.4	(22.3, 62.6)	43.9	(38.8, 49.1)	38.2	(33.0, 43.5)
**BMI**																
Underweight	19.8	(17.2, 22.5)	14.7	(12.4, 16.9)	25.7	(20.5, 31.0)	18.9	(14.1, 23.8)	21.5	(7.5, 35.3)	15.1	(3.3, 26.9)	25.1	(20.2, 30.4)	18.6	(14.0, 23.3)
Normal	30.0	(28.1, 31.8)	25.0	(23.3, 26.8)	41.0	(37.9, 44.2)	34.9	(31.8, 38.0)	43.5	(23.9, 63.1)	41.7	(22.8, 60.7)	41.3	(37.7, 45.0)	35.7	(32.1, 39.2)
Overweight	43.7	(40.7, 46.9)	40.0	(36.6, 42.6)	55.5	(50.2, 60.6)	50.0	(44.7, 55.2)	44.3	(22.6, 66.0)	41.3	(20.7, 61.9)	53.1	(46.9, 59.2)	48.2	(42.3, 53.5)
Obese	54.0	(49.5, 58.5)	49.3	(44.8, 54.0)	66.7	(58.1, 75.2)	60.6	(52.1, 69.2)	66.9	(36.4, 97.4)	59.2	(31.5, 86.8)	67.4	(60.3, 74.8)	61.5	(54.1, 69.0)
**Tobacco usage**																
Yes	28.6	(26.3, 30.8)	23.5	(21.5, 25.9)	36.0	(32.5, 39.4)	28.4	(25.1, 31.6)	43.8	(23.6, 64.3)	38.9	(20.6, 57.7)	37.6	(33.8, 41.4)	30.2	(26.5, 34.0)
No	36.3	(34.1, 38.5)	31.6	(30.0, 33.6)	49.5	(45.7, 53.1)	44.2	(40.3, 47.9)	42.1	(22.7, 61.4)	3906	(21.3, 58.0)	48.1	(43.6, 52.6)	43.3	(38.8, 47.6)
**Alcohol usage**																
Yes	27.4	(25.0, 30.9)	23.0	(20.8, 25.7)	38.4	(34.5, 46.4)	29.7	(25.0, 34.4)	37.7	(18.6, 51.9)	35.7	(17.8, 53.9)	38.9	(33.7, 44.3)	30.7	(25.7, 35.5)
No	35.0	(33.1, 36.9)	30.0	(28.3, 31.4)	45.7	(42.3, 49.4)	40.3	(36.6, 43.5)	44.1	(24.9, 60.3)	40.3	(22.0, 58.5)	45.4	(41.2, 49.5)	40.2	(36.1, 44.3)
**Heart Disease**																
No	32.4	(30.7, 34.0)	27.7	(26.5, 30.0)	43.1	(40.0, 46.2)	37.1	(33.8, 40.3)	34.0	(22.1, 57.8)	36.6	(20.2, 53.2)	42.8	(39.2, 46.3)	37.3	(33.7, 40.5)
Yes	43.5	(38.2, 48.9)	37.4	(33.0, 42.8)	58.2	(49.1, 67.3)	50.8	(41.1, 60.4)	64.4	(34.8, 93.4)	60.2	(31.7, 88.8)	59.4	(50.6, 68.1)	53.0	(43.2, 61.9)
**Diabetes**																
No	28.1	(26.5, 29.7)	23.4	(22.0, 24.8)	37.3	(35.0, 39.6)	31.1	(28.9, 33.3)	37.7	(20.3, 55.0)	34.6	(18.3, 50.9)	37.6	(34.5, 40.6)	31.7	(28.8, 35.0)
Yes	65.9	(62.7, 69.1)	60.6	(57.3, 63.9)	76.4	(70.2, 82.5)	70.6	(64.4, 76.8)	82.3	(59.6, 100.0)	75.1	(49.8, 100.0)	76.2	(70.0, 82.4)	70.3	(63.9, 76.6)

Refer to S1 Table.

### Socio-economic characteristics of hypertension by stages

**Table 4** presents the relative risk ratios (RRR) for various socioeconomic and demographic factors associated with different stages of hypertension, using optimal hypertension as the reference category. Age was a significant factor across all stages of hypertension, with the risk of stage 1 (RRR = 2.24, p < 0.001) and stage 2 (RRR = 3.44, p < 0.001) hypertension being notably higher in the 75 + years age group compared to the 45–54 years age group (stage 1: RRR = 1.45, p < 0.001; stage 2: RRR = 1.69, p < 0.001). Females had a lower risk of stage 1 hypertension (RRR = 0.88, p = 0.018) and stage 2 hypertension (RRR = 0.76, p = 0.002) compared to males, indicating a gender difference in severe hypertension risk. Urban residents had a higher risk of stage 1 hypertension (RRR = 1.15, p = 0.046) compared to rural residents. The ST older adults had a significantly higher risk of stage 2 hypertension (RRR = 1.62, p < 0.001) compared to the SC categories. The older adults who were living with children and others had significantly less risk of stage 1 hypertension (RRR = 0.71, p = 0.002) and stage 2 hypertension (RRR = 0.64, p < 0.001) as compared to those living alone. Body Mass Index (BMI) was a critical factor, with overweight and obese individuals having significantly higher risks of all stages of hypertension. For instance, obese individuals had an RRR of 6.56 (p < 0.001) for stage 2 hypertension and a RRR of 5.05 (p < 0.001) for stage 1 hypertension. The older adults who had diabetes had a significantly higher risk of stage 1 hypertension (RRR = 1.44, p < 0.001) and stage 2 hypertension (RRR = 1.77, p < 0.001) compared to those without diabetes.

**Table 4:** Relative risk ratios from multinomial logistic regression for hypertension stages among older adults in India, 2017−18.

Socio-economic characteristics (Ref: Optimal)	Pre-Hypertensive			Stage 1 (Grade 1)			Stage 2 (Grade 2/ Grade 3)		
	RRR	p-value	95% CI	RRR	p-value	95% CI	RRR	p-value	95% CI
**Age**									
45-54	**Ref.**			**Ref.**			**Ref.**		
55-64	1.21	0.000	(1.12, 1.32)	1.45	0.000	(1.29, 1.63)	1.69	0.000	(1.33, 2.14)
65-74	1.52	0.000	(1.33, 1.75)	2.16	0.000	(1.92, 2.43)	2.28	0.000	(1.87, 2.79)
75+	1.58	0.000	(1.33, 1.89)	2.24	0.000	(1.91, 2.64)	3.44	0.000	(2.64, 4.49)
**Sex**									
Male	**Ref.**			**Ref.**			**Ref.**		
Female	0.81	0.000	(0.74, 0.89)	0.88	0.018	(0.78, 0.98)	0.76	0.002	(0.64, 0.91)
**Place of residence**									
Rural	**Ref.**			**Ref.**			**Ref.**		
Urban	1.11	0.050	(1.00, 1.24)	1.15	0.046	(1.00, 1.32)	1.08	0.336	(0.92, 1.27)
**MPCE quintile**									
Poorest	**Ref.**			**Ref.**			**Ref.**		
Poorer	0.96	0.477	(0.87, 1.06)	0.98	0.771	(0.87, 1.11)	0.99	0.868	(0.85, 1.15)
Middle	1.02	0.750	(0.92, 1.12)	0.98	0.765	(0.87, 1.11)	1.01	0.886	(0.85, 1.21)
Richer	0.88	0.047	(0.77, 1.00)	0.82	0.029	(0.69, 0.98)	0.92	0.454	(0.73, 1.15)
Richest	0.96	0.470	(0.86, 1.07)	0.92	0.192	(0.81, 1.04)	0.98	0.909	(0.74, 1.30)
**Caste**									
SC	**Ref.**			**Ref.**			**Ref.**		
ST	1.38	0.000	(1.20, 1.58)	1.33	0.000	(1.14, 1.55)	1.62	0.000	(1.32, 2.00)
OBC	1.00	0.953	(0.90, 1.11)	0.93	0.246	(0.82, 1.05)	1.08	0.304	(0.93, 1.24)
Others	1.12	0.031	(1.01, 1.23)	1.09	0.203	(0.96, 1.24)	1.18	0.048	(1.00, 1.39)
**Religion**									
Hindu	**Ref.**			**Ref.**			**Ref.**		
Muslim	1.00	0.965	(0.86, 1.15)	1.25	0.001	(1.10, 1.42)	0.99	0.935	(0.83, 1.19)
Christians	0.86	0.344	(0.64, 1.17)	0.88	0.341	(0.67, 1.15)	1.15	0.373	(0.84, 1.57)
Others	1.07	0.435	(0.90, 1.26)	1.29	0.003	(1.09, 1.53)	1.64	0.000	(1.26, 2.15)
**Education level**									
No education	**Ref.**			**Ref.**			**Ref.**		
Less than 5 years	1.08	0.252	(0.95, 1.23)	1.14	0.037	(1.01, 1.30)	0.96	0.648	(0.80, 1.15)
5–9 years	1.03	0.578	(0.93, 1.13)	1.08	0.186	(0.96, 1.21)	0.95	0.606	(0.78, 1.15)
10 years or more	1.02	0.776	(0.88, 1.19)	1.01	0.928	(0.80, 1.28)	0.93	0.484	(0.77, 1.13)
**Marital Status**									
Currently Married	**Ref.**			**Ref.**			**Ref.**		
Widowed	0.91	0.534	(0.67, 1.23)	1.09	0.618	(0.78, 1.53)	1.45	0.109	(0.92, 2.27)
Others	0.59	0.013	(0.38, 0.89)	0.69	0.109	(0.44, 1.08)	1.09	0.766	(0.63, 1.87)
**Living Arrangement**									
Living alone	**Ref.**			**Ref.**			**Ref.**		
Living with spouse and/or others	0.47	0.000	(0.32, 0.68)	0.52	0.001	(0.34, 0.78)	0.61	0.055	(0.36, 1.01)
Living with spouse and children	0.47	0.000	(0.33, 0.66)	0.49	0.000	(0.33, 0.72)	0.61	0.046	(0.37, 0.99)
Living with children and/or others	0.65	0.000	(0.53, 0.81)	0.71	0.002	(0.57, 0.88)	0.64	0.000	(0.50, 0.81)
**Working Status**									
Currently working	**Ref.**			**Ref.**			**Ref.**		
Ever worked	1.04	0.325	(0.96, 1.14)	1.05	0.371	(0.94, 1.17)	1.20	0.007	(1.05, 1.37)
Never worked	1.04	0.408	(0.94, 1.15)	0.92	0.213	(0.81, 1.05)	0.97	0.759	(0.80, 1.18)
**Health Insurance**									
No	**Ref.**			**Ref.**			**Ref.**		
Yes	1.07	0.135	(0.98, 1.16)	1.06	0.181	(0.97, 1.16)	1.12	0.171	(0.95, 1.31)
**BMI**									
Underweight	**Ref.**						**Ref.**		
Normal	1.88	0.000	(1.73, 2.05)	2.35	0.000	(2.14, 2.59)	2.33	0.000	(2.02, 2.67)
Overweight	3.25	0.000	(2.80, 3.78)	5.02	0.000	(4.33, 5.80)	5.85	0.000	(4.62, 7.42)
Obese	3.34	0.000	(2.54, 4.39)	5.05	0.000	(4.16, 6.13)	6.56	0.000	(5.08, 8.48)
**Tobacco usage**									
Yes	**Ref.**			**Ref.**			**Ref.**		
No	1.04	0.385	(0.96, 1.12)	1.07	0.169	(0.97, 1.18)	1.12	0.107	(0.98, 1.29)
**Alcohol usage**									
Yes	**Ref.**			**Ref.**			**Ref.**		
No	0.92	0.130	(0.82, 1.03)	0.67	0.000	(0.59, 0.75)	0.64	0.000	(0.54, 0.76)
**Heart Disease**									
No	**Ref.**			**Ref.**			**Ref.**		
Yes	0.96	0.459	(0.86, 1.07)	1.10	0.175	(0.96, 1.27)	1.04	0.709	(0.84, 1.29)
**Diabetes**									
No	**Ref.**			**Ref.**			**Ref.**		
Yes	1.38	0.000	(1.16, 1.64)	1.44	0.000	(1.27, 1.65)	1.77	0.000	(1.39, 2.26)

(reference category: Optimal hypertension); Refer to S1 Table.

**Fig 3:**
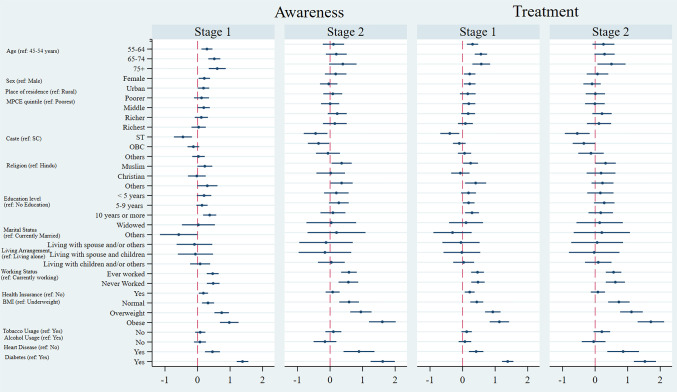
Average marginal effects on probability of awareness and treatment of hypertension by stages among older adults in India, 2017−18.

**Fig 3** presents the average marginal effects (AME) on the probability of awareness and treatment of hypertension among older adults in India, stratified by stages of hypertension. For grade 1 hypertension, the probability of awareness was significantly higher among females, urban residents, as well as those having diabetes or heart disease, than their respective counterparts. Educational levels, particularly those with 10 or more years of schooling, also showed a positive correlation with awareness. For stage 2 hypertension, similar trends were observed, with stronger effects for urban residents, higher economic status, and co-morbidities. The AME for obesity was significant and positive for both stage 1 and stage 2 hypertension, and obesity was associated with increased hypertension awareness. Older age groups (65–74 years and 75 + years) demonstrated higher treatment probabilities compared to the 55–64 years age group. Hypertension treatment was higher among females, indicating that they were more likely to receive treatment for hypertension than males. Urban residence showed a significant positive effect on treatment as compared to rural counterparts. The presence of diabetes or heart disease significantly increases the likelihood of receiving hypertension treatment. Overweight and obesity showed positive associations with treatment probability.

### Age at and duration of hypertension onset by stage

**Table 5** shows the age and duration of onset of hypertension by stages among older adults in India. The average age at onset of hypertension among older adults was 56 years, with an interquartile range (IQR) between 48 and 64. Optimal blood pressure had its onset at the mean age of 54 years, pre-hypertension at 55 years, Stage 1 hypertension (grade 1) at 56 years, grade 2 hypertension at 57 years, grade 3 hypertension starts at the mean age of 56 years, and stage 2 hypertension (grade 2/grade 3) at 57 years. The overall duration of hypertension was 84 months, with a median of 60 months and an IQR ranging from 24 to 120 months. The duration of hypertension varied slightly across stages as follows: 79 months for optimal, 86 months for pre-hypertension, 85 months for stage 1 hypertension (grade 1), 83 months for grade 2 hypertension, and 79 months for grade 3 hypertension, and 83 months for stage 2 hypertension (grade 2/ grade 3).

**Table 5:** Age and duration of onset of hypertension by stages among older adults in India, 2017−18.

Stages of hypertension	Mean	95% CI	Median	IQR
***Age at onset (in years)***				
Overall	56	(56, 57)	56	(48, 64)
Optimal	54	(54, 55)	52	(44, 61)
Pre-hypertensive	55	(54, 56)	54	(46, 63)
Stage 1 (Grade 1)	56	(55, 57)	55	(47, 63)
Grade 2	57	(56, 58)	56	(48, 64)
Grade 3	56	(52, 60)	54	(45, 64)
Stage 2 (Grade 2/ Grade 3)	57	(56, 58)	54	(45, 64)
***Duration (in Months)***				
Overall	84	(78, 90)	60	(24, 120)
Optimal	79	(72, 85)	48	(24, 108)
Pre-hypertensive	86	(71, 101)	60	(24, 108)
Stage 1 (Grade 1)	85	(81, 90)	60	(24, 108)
Grade 2	83	(77, 89)	60	(24, 120)
Grade 3	79	(63, 94)	60	(24, 120)
Stage 2 (Grade 2/ Grade 3)	83	(77, 88)	60	(24, 120)

Refer to SM S1 Table.

### Socio-economic inequalities in hypertension care cascade

**Fig 4:**
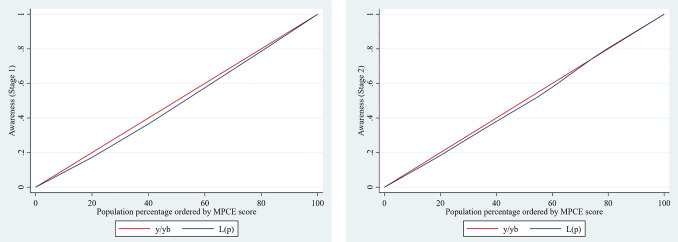
Concentration curve for hypertension awareness by (A) Stage 1 and (B) Stage 2 among older adults in India, 2017−18.

**Fig 5:**
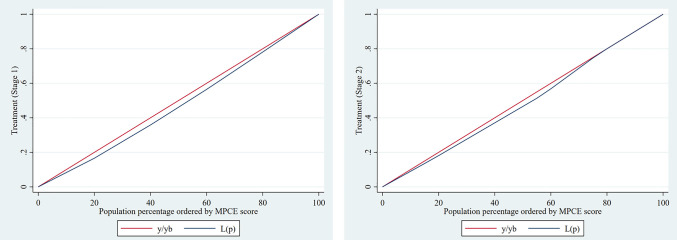
Concentration curve for hypertension treatment by (A) Stage 1 and (B) Stage 2 among older adults in India, 2017−18.

**Table 6** shows the results of the ECI on awareness and treatment of hypertension by stages in India. In the case of stage 1 hypertension (grade 1), the ECI for prevalence was 0.014 and not significant. The ECI values at stage 1 hypertension for awareness and treatment were both statistically significant at 0.052 and 0.058, respectively, indicating a higher concentration of awareness and treatment among the wealthy. With regard to stage 2 hypertension, the values of ECI for prevalence were statistically significant (0.014), but the ECI values at 0.052 and 0.063 for awareness and treatment were not statistically significant, although higher concentration among richer individuals (Figs 4 and 5).

**Table 6:** Erreygers’ concentration index of prevalence, awareness and treatment of hypertension by stages among older adults in India, 2017−18.

Stages of hypertension	Variables	N	ECI	95% CI	p-value
Stage 1 (Grade 1)	Prevalence	58,787	0.014	(−0.009, 0.037)	0.222
	Awareness	13,945	0.052	(0.019, 0.085)	0.002
	Treatment	13,945	0.058	(0.027, 0.089)	0.000
Grade 2	Prevalence	58,787	0.006	(−0.007, 0.019)	0.361
	Awareness	5,272	0.096	(0.032, 0.159)	0.003
	Treatment	5,272	0.097	(0.030, 0.164)	0.004
Grade 3	Prevalence	58787	0.008	(−0.009, 0.025)	0.345
	Awareness	694	−0.198	(−0.576, 0.180)	0.184
	Treatment	694	−0.176	(−0.531, 0.180)	0.333
Stage 2 (Grade 2/ Grade 3)	Prevalence	58787	0.014	(−0.001, 0.029)	0.063
	Awareness	5,966	0.059	(−0.039, 0.157)	0.237
	Treatment	5,966	0.063	(−0.030, 0.158)	0.180

Refer to S1 Table.

## Discussion

The stage of hypertension is a marker of vulnerability to cardiovascular diseases and other health complications. Various health programs have identified different stages of hypertension for prevention and care. This study provides insights into the hypertension care cascade among older adults in India by estimating prevalence, awareness, and treatment across different stages of hypertension. Our salient findings are as follows.

First, we found a high overall prevalence, with approximately two-fifths of adults classified as being pre-hypertensive, over one-fifth as having stage 1 hypertension, and one-tenth as having stage 2 hypertension. Second, our findings revealed substantial gaps in awareness and treatment, particularly among individuals at advanced hypertension stages (grade 2 and grade 3), highlighting a severe public health concern in the country. Only around 42% of individuals with grade 3 hypertension, the most severe stage, were aware of their condition, and even fewer (38%) were receiving treatment. The awareness and treatment rates were relatively higher in stage 2 than stage 1. Third, our age-sex adjusted estimates suggested a strong socio-economic gradient in awareness and treatment of hypertension across all stages among the rich and the educated. The concentration index for awareness and treatment was pro-rich across all stages. Fourth, increasing age was significantly associated with higher odds of hypertension, while living with spouses and children was associated with a lower chance of hypertension across all stages. Body mass index (BMI), alcohol use, and diabetes were significant predictors across stages. The economic condition of the household, educational attainment, and social group were not significant predictors across stages/ grades.

Our findings on the pattern and cascade of hypertension care are consistent with the literature. Since we followed the Government of India classification of stages of hypertension, and specifically for older adults 45 + , which are not directly comparable to other studies, we found similar socio-economic patterns as observed elsewhere. A study from Bangladesh found that 26.5% of the surveyed individuals had pre-hypertension, 12.1% had stage 1 hypertension, and 7% had stage 2 hypertension in rural regions and that the prevalence rates of all the stages of hypertension were higher in the urban areas than the rural regions [30]. A study done in two countries of Sub-Saharan Africa, namely South Africa and Ghana, found the weighted prevalence of stage 1 and stage 2 hypertension to be 18.5% and 28.1% each in South Africa and 21.6% each in Ghana. Similar to our findings, the prevalence was found to increase with age at varying stages in these two countries [31]. The estimates of stage 1 and stage 2 hypertension are much lower in developed countries such as Canada, the USA, and the UK.

Our finding that socio-economic factors do not consistently predict hypertension across stages is consistent with the literature. A study done in Ghana and South Africa found that age, income, and BMI were the independent predictors for stage-1 hypertension [31]. Another study found that men had a significantly lower probability of hypertension awareness than women at stage 1 but not at stage 2 [32]. In Taiwan, the progression from pre-hypertension to stage 1 hypertension was positively associated with males, higher waist circumference, and having parents with hypertension [33].

Consistent with prior studies, our findings indicated a substantial gap in awareness and treatment across all hypertension stages [9,13]. This scenario suggests significant missed opportunities for early detection and timely intervention, exposing many older adults to an increased risk of cardiovascular complications [23,34,35]. Socioeconomic disparities were evident, with higher awareness and treatment among wealthier, educated, and urban residents than their respective counterparts. This aligns with the global evidence that indicates a persistent socioeconomic gradient in hypertension care [12,14]. The mean age of onset for all stages was the mid-50s, with a slightly higher onset age for grade 2 hypertension (57 years). The relatively long duration between onset and diagnosis for more severe stages requires more frequent and comprehensive health check-ups among older adults. These findings are consistent with the global trend of hypertension onset in middle age and delayed diagnosis in many LMICs [16,36].

Our findings of high prevalence of stage 2 hypertension (both grade 2 and grade 3) hypertension are possibly due to the medical condition remaining undiagnosed. A large proportion of the population was never diagnosed or aware of its condition, so they did not seek treatment. This led to a large socio-economic inequality in the awareness and treatment of hypertension across stages and to the pro-rich nature of hypertension. Our findings of economic condition and educational attainment not being significant predictors suggest that the disease is now common in Indian households.

While our study provides valuable insights, it is important to acknowledge certain limitations. First, the cross-sectional nature of the LASI data limited our ability to establish causal relationships between socioeconomic factors and hypertension outcomes. Second, our reliance on self-reported data for awareness and treatment may have introduced recall bias, potentially leading to under or overestimating these parameters. Third, we did not consider those who were under medication and had their hypertension in control in our analytical sample. Fourth, our study used the Government of India classification of hypertension by stages, which might not be representative of the international cut-offs.

## Conclusion

In conclusion, our study revealed that even severe hypertension cases remained largely undiagnosed and untreated at advanced stages, indicating significant missed opportunities for early intervention. The low levels of awareness and treatment, especially at the advanced stages of hypertension, show the urgent need for robust screening programs targeting high-risk populations. Addressing socioeconomic and educational inequalities in hypertension care is essential. Public health initiatives focusing on health literacy, affordable healthcare access, and community-based management could substantially reduce disparities in hypertension management. To mitigate the significant urban-rural divide in hypertension awareness and treatment, steps like scaling up rural healthcare infrastructure and improving service delivery mechanisms are needed. The high prevalence of stage 1 needs early intervention and strategies to prevent progression to more severe stages.

## Supporting information

S1 Table Classification of hypertension stages by cut-off’s of SBP and DBP according to different guidelines. (PDF)
