# Supplementary material for: Care cascade of hypertension across stages among older adults in India
Source: PLoS One. 2025 Dec 3;20(12):e0335627. doi: 10.1371/journal.pone.0335627 (PMC12674583; doi:10.1371/journal.pone.0335627)
Supplement: S1 Table — (PDF) [file pone.0335627.s001.pdf]

**Table S1: Classification of Hypertension stages by cut-off's of SBP and DBP according to different guidelines**

| Category | Systolic (mmHg) | Diastolic (mmHg) |
|---|---|---|
| **In this paper** | | |
| Optimal (Normal) | <120 | <80 |
| Pre-hypertension (Normal+ High Normal) | 120 to 139 | 80 to 89 |
| Stage 1 hypertension (Grade 1 ) | 140 to 159 | 90 to 99 |
| Stage 2 hypertension (Grade 2 and Grade 3) | ≥160 | ≥100 |
| **Government of India** | | |
| Optimal | <120 | <80 |
| Normal | 120 to 129 | 80 to 84 |
| High Normal | 130 to 139 | 85 to 89 |
| Grade 1 hypertension | 140 to 159 | 90 to 99 |
| Grade 2 hypertension | 160 to 179 | 100 to 109 |
| Grade 3 hypertension | ≥ 180 | ≥ 110 |
| **International Society of Hypertension** | | |
| Normal | <120 | <80 |
| Prehypertension | 120 to 139 | 80 to 89 |
| Stage 1 hypertension | 140 to 159 | 90 to 99 |
| Stage 2 hypertension | ≥ 160 | ≥ 100 |
| **European Society of Hypertension** | | |
| Optimal | <120 | <80 |
| Normal | 120 to 129 | 80 to 84 |
| High Normal | 130 to 139 | 85 to 89 |
| Grade 1 hypertension | 140 to 159 | 90 to 99 |
| Grade 2 hypertension | 160 to 179 | 100 to 109 |
| Grade 3 hypertension | ≥ 180 | ≥ 110 |
| **Eigth Joint National Committee** | | |
| Normal | <120 | <80 |
| Prehypertension | 120 to 139 | 80 to 89 |
| Stage 1 hypertension | 140 to 159 | 90 to 99 |
| Stage 2 hypertension | ≥ 160 | ≥ 100 |

***Note:*** *Optimal blood pressure is considered as normal, because the level of blood pressure at below 120/80 mmHg, indicates the healthy function of blood vessel and no cardiovascular risk.;*
*Pre-hypertension is considered as the combination of normal and high normal, because the range of blood pressure is higher than the optimal i.e. 120-139/ 80-89 mmHg. It indicates the blood pressure reading is normal and threshold for hypertension, which increases the cardiovascular risk.*

**Reference**

Dubey, M., Rastogi, S., & Awasthi, A. (2019). Hypertension prevalence as a function of different guidelines, India. *Bulletin of the World Health Organization*, *97*(12), 799.
